# Feasibility of Microlearning for Improving the Self-Efficacy of Cancer Patients Managing Side Effects of Chemotherapy

**DOI:** 10.1007/s13187-023-02324-6

**Published:** 2023-07-14

**Authors:** Anna Janssen, Kavisha Shah, Melanie Rabbets, Adnan Nagrial, Christopher Pene, Clare Zachulski, Jane L. Phillips, Paul Harnett, Tim Shaw

**Affiliations:** 1 Faculty of Medicine and Health, The University of Sydney, Sydney, Australia; 2 Crown Princess Mary Cancer Centre, Western Sydney Local Health District, Westmead, NSW Australia; 3 Sacred Heart Service, St Vincent’s Hospital, Sydney, NSW Australia; 4 Faculty of Health, School of Nursing, Queensland University of Technology, QLD, Brisbane, Australia; 5 IMPACCT, Faculty of Health, University of Technology Sydney, Ultimo, Australia

**Keywords:** Patient education, Lung cancer, Online learning, Digital health

## Abstract

Lung cancer patients have a high symptom burden that negatively affects their quality of life. Increasing patient self-efficacy to deal with treatment side effects can ameliorate their symptom burden. Education programs can help enhance patient self-efficacy by giving patients more control over their condition through increased disease literacy. This study aimed to evaluate the feasibility of microlearning for delivering lung cancer patients’ information on side effects of chemotherapy. Secondary objectives of the program are to understand the acceptability of microlearning for delivery this type of education to lung cancer patients and the potential impact of microlearning on patient self-efficacy, knowledge and confidence managing side effects of chemotherapy. A mixed-methods prepost test (or quasi-experimental) study design was used to better enable patients to identify and manage the side effects of their condition and chemotherapy. Participants were patients diagnosed with stage II to stage IV lung cancer, who had a life expectancy of greater than 3 months and were aged 18 years or older. Multiple validated scales were used to assess patient self-efficacy pre- and post-intervention. The online program was evaluated using quantitative data of completion rates extracted from the online platform. Semi-structured interviews were used to explore the impact of the online program on perceived self-efficacy and quality of life. Twenty-three participants agreed to participate in the study and five agreed to complete a semi-structured interview. Participants found the content comprehensive, relevant and engaging. The program improved perceived disease literacy and helped participants develop coping strategies to manage side effects. Participants also found the platform easy to use and navigate. Additional courses and features were requested. Patients with a diagnosis of cancer receive a large amount of information about the side effects of chemotherapy and how to manage them. This information is often provided soon after diagnosis or upon commencement of therapy, which can be overwhelming for some patients. Microlearning, a method of online learning that spaces distributing of content over several weeks, may be a useful tool for supporting delivering of health information to this group of patients.

## Introduction

Globally, lung cancer is the second most commonly diagnosed cancer, with around 2.21 million new cases in 2020 [1]. It remains the most burdensome cancer type for patients and healthcare systems alike [2], with an overall survival rate of only 16.8% [3]. Most patients are diagnosed at an advanced stage and experience multiple symptoms, often simultaneously, because of their disease and/or treatment(s). Lung cancer symptom ‘clusters’ include fatigue, breathlessness, pain, distress, nausea and vomiting [4]. Appropriate management is important as unrelieved symptoms negatively impact patients’ psychosocial health and quality of life [4]. Inversely, psychosocial symptoms, such as sadness and anxiety, can increase fatigue and pain frequency and severity [5]. The positive feedback loop between symptom clusters and emotional distress remains largely unaddressed with physical and psychological unmet needs more frequently reported by patients with advanced cancer [6]. Greater patient involvement in symptom management can help alleviate the disruptive effect of this feedback loop on quality of life [7].

Good self-management can be achieved by increasing patient self-efficacy. Higher self-efficacy levels can help reduce symptom distress, enable greater disease adjustment and enhance satisfaction with cancer care [8]. Factors that affect self-efficacy include health literacy and active involvement in medical decision-making. However, patients need to understand the required health behaviours and have the confidence to perform them to successfully manage their condition [9]. This is concerning as a delayed lung cancer diagnosis is often attributed to a lack of disease and symptom awareness [10]. The impact of low health literacy is realised through decreased participation in treatment decision-making as patients are unable to fully communicate their concerns to their cancer care team or understand their treatment goals [8]. Consequently, patients with lung cancer could be less equipped to manage the side effects of their treatment.

Patients from culturally and linguistically diverse (CALD) backgrounds face additional barriers to effective symptom self-management. These patients are more likely to be diagnosed at an advanced stage and consequently have higher mortality rates than non-CALD patients [11]. Poorer patient outcomes among this cohort can be attributed to multiple factors including health literacy, poor health communication and fatalism [12]. Prior research has found that providers are more verbally dominant, hurried and spend less time building relationships with CALD patients when compared to their White counterparts [13]. These communication inequalities discourage active participation in medical decision-making [14]. Combined, these barriers lower health services utilisation and decrease symptom awareness in CALD populations.

One way to encourage self-management is through patient education programs. Education programs enable patients to have greater control over side effects by increasing disease literacy and symptom awareness [15]. These programs are commonly delivered face-to-face, but often patients cannot attend due to their cancer, its treatment or logistics (e.g. travel, cost) [16]. This is particularly concerning as lower socioeconomic status has been associated with a higher incidence of lung cancer [17]. Access can be improved by delivering programs online. Online programs have been used effectively with patients with various cancer types, including lung, prostate and breast cancer, as well as across different age groups and cultural backgrounds [10]. The impact of online programs can be further enhanced by involving consumer representatives during development given the emphasis on patient autonomy in self-management [8].

This study aimed to evaluate the feasibility of microlearning for delivering lung cancer patients’ information on side effects of chemotherapy. Secondary aims of the program are to understand the acceptability of microlearning for delivery this type of education to lung cancer patients and the potential impact of microlearning on patient self-efficacy, knowledge and confidence managing side effects of chemotherapy.

## Methodology

### Study Design

A mixed methods quasi-experimental study [18]. This design was selected as being the most suitable to evaluating both the feasibility and acceptability of an online microlearning program.

### Study Setting, Participants and Intervention Design

People with advanced lung cancer attending an oncology department at a public metropolitan hospital in Sydney, Australia. Around half the population serviced by this hospital are born overseas and/or speak a language other than English at home.

Eligible participants were patients diagnosed with stage II to stage IV lung cancer, who had a life expectancy of greater than 3 months and were aged 18 years or older. Participants also needed access to an internet connection and a level of spoken and written English proficiency that would enable them to understand the online education content. Participants who did not meet one or more of these requirements or were too physically or mentally unwell to participate were excluded. Potential participants were approached and assessed for eligibility by a clinical nurse consultant.

### Intervention

The online patient education program was developed using an online microlearning platform that sent multiple choice cases to participants via email. Microlearning is a branch of online learning that is context-based and delivers short, focused lessons in a recurring and spaced manner [19]. Microlearning or spaced learning offers a number of advantages compared to more ‘traditional’ types of online learning including the ability to have learners engage more frequently with the material; chunks content into digestible pieces of information; allows for a high degree of flexibility and is easily accessible.

The microlearning platform spaces delivery of cases so that learners only receive them in bundles of one or two every few days. The delivery of the cases is spaced out every few days until a learner has seen all the cases at least once. Spacing of cases is used by the platform to minimise the time required to answer each case, with only 5 to 10 min required for each bundle. The platform then repeats the cases a set number of times depending on whether participants selected correct or incorrect responses. Cases are retired when the learner has answered all required repeats, and the program is complete when all cases have been retired. In this intervention, each case provided an example of a common side effect that could be experienced whilst undergoing chemotherapy and advice on how to self-manage that side effect. Participants received the feedback once a response to the case was selected, correct or incorrect. The case feedback also provided contact information for the cancer centre so that participants could get advice from the healthcare team on side effect management, if required.

Eight lung cancer symptom self-management questions were developed by the research team in collaboration with a multidisciplinary advisory committee who had experience in medical oncology, nursing and palliative care. Questions addressed the identification and management of eight common lung cancer symptoms or experiences (i.e. pain, breathlessness, fatigue and pain management). Once the cases were developed, there were reviewed by a health literacy expert, to ensure clarity of content for the study participants. Subsequently two lung cancer patients reviewed the cases to confirm the appropriateness of the content and its presentation in the case. The final version of the online cases was completed in February 2020, and they were subsequently uploaded to the microlearning platform.

The intervention was administered to each participant over 8 weeks between mid-March 2019 and early December 2020. During the intervention period, participants were also asked to use a structured symptom diary to capture incidence of side effects from chemotherapy occurring during the intervention.

### Data Collection and Analysis

Quantitative data collected included responses to baseline and exit surveys as well as metrics from the online learning platform used to deliver the intervention. Qualitative data consisted of semi-structured interviews, symptom diary records and open-ended responses in the exit survey. Quantitative data was analysed using SPSS statistical analysis software (IBM Corp). Data was analysed for differences in self-efficacy for participants who completed both pre-and post-surveys. Qualitative data was analysed thematically by two researchers (AJ and KS) to identify initial codes and categorise findings into final themes.

Quantitative data included:

1. Metrics captured by the online learning platform on participant progress through the course and responses to validated surveys. Metrics routinely collected by the online platform were extracted to evaluate program use. This included the number of questions participants were enrolled in that they completed and the time elapsed between being allocated a question and answering it.
2. Validated surveys were administered 2 weeks prior and 2 weeks post-intervention. Baseline data consisted of three validated scales used to assess participant self-efficacy: ‘Self-Efficacy for Managing Chronic Diseases 6-item Scale’ [20], perceived involvement in care: ‘Patients’ Perceived Involvement in Care Scale’ [21] and technological literacy ‘Online Technologies Self-Efficacy Scale’ [22]. The technological literacy scale consisted of three subscales: (1) Internet Competencies subscale; (2) Synchronous Interaction subscale and (3) Asynchronous Interaction subscale.
3. Post-intervention the self-efficacy scale and the perceived involvement in care scale were repeated.

Qualitative data consisted of:

1. Semi-structured interviews were conducted to evaluate the program and the impact of the intervention on perceived patient self-efficacy. This data was thematically analysed to identify emergent and overarching themes and exemplar quotes for each theme. Two researchers independently read the first two transcripts and created draft codes. Differences were resolved in discussion with the research team, whilst additional transcripts were coded by a single researcher. New themes or subthemes were discussed to iteratively refine the draft coding scheme.
2. Symptom diary records to determine the influence of the intervention on symptom management as well as the feasibility of the diary as a measurement tool. Participants were asked to rate the severity of four symptoms on a scale from 1 to 5 daily for 10 weeks: pain, nausea/vomiting, breathlessness and constipation. Participants were also asked to report whether they sought professional help for each symptom each week with a free-text box for further information.
3. Free-text responses collected in the exit survey asking participants for program feedback were completed approximately 6 weeks post-intervention. The survey consisted of fifteen Likert scale questions and three open-response questions.

As a mixed methods study was undertaken, data was integrated to understand key insights from the results. Data from different sources was triangulated to build a more complete picture of the strengths and weaknesses of the course, and develop a nuanced understanding of how the program could be refined to align more closely with patient needs in future.

### Ethics

Permission to conduct this study was granted by human research ethics committee of the Western Sydney Local Health District (Protocol No 4455).

## Results

### Quantitative Data

#### Demographic and Microlearning Platform Engagement Metrics

A total of 24 patients consented to participate in the program. One participant subsequently withdrew consent during the study and data collected about them was excluded from analysis. Of these 23 participants who consented to participant, 20 provided information on their age as part of the consenting process. These participants ranged in age from 46 to 73, with a mean age of 62. Only 22 participants could be enrolled in the online program, as one participant email did not work when it was used to register them in the online program.

Of the participants who were enrolled in the program, 19 started the program (86.36%) and 14 subsequently completed it (73.68%). Two of the participants who started but did not finish the program answered more than half of the questions. All program completers were scored by the platform as being ‘Very Active’, which indicated that they answered each question within 2 days of receiving it.

#### Pre-intervention Validated Surveys

##### Digital Literacy of Participants [22]

Ten out of eleven participants completed the Internet Competencies subscale. Most participants feel very confident (50%) or somewhat confident (30%) opening a web browser. All participants feel very confident (50%) or somewhat confident (50%) reading text from a website. Similarly, the majority of participants feel very confident (50%) or somewhat confident (40%) clicking on a link to visit a specific website. Most participants also feel very confident (40%) or somewhat confident (30%) accessing a specific website by typing the address or URL.

Likewise, most participants feel very confident (60%) or somewhat confident (30%) conducting an internet search using one or more keywords. The majority of participants also feel very confident (40%) and somewhat confident (30%) printing a website. In contrast, most participants are not very confident (60%) bookmarking a website or copying a block of text from a website and pasting it to a document in a word processor. The majority of participants are also not very confident (50%) or somewhat confident (30%) with downloading (saving) an image from a website to a disc.

Ten out of eleven participants completed the Synchronous Interaction subscale. Most participants feel not very confident (60%) providing a nicking, reading messages from one or more members, or interacting privately with one member of a synchronous chat system. The majority of participants are also not very confident (50%) or somewhat confident (20%) answering a message or providing my own message in a chat system (a one-to-many interaction).

All participants completed the Asynchronous Interaction subscale. Most participants feel not very confident (36%) or somewhat confident (46%) significant on and off an asynchronous conferencing system or posting a new message to a synchronous conferencing system. Majority of participants are also not very confident replying to a message posted on an asynchronous conferencing system so that only one member can view it (reply to sender). In contrast, the majority of participants are somewhat confident (46%) or very confident (27%) reading a message posted on an asynchronous conferencing system or replying to a message posted on an asynchronous conferencing system so that all members can view it. In addition, most participants (73%) are not very confident downloading a file from an asynchronous conferencing system to a local disc. Likewise, the majority of participants do feel not very confident (55%) uploading (sending) a file to an asynchronous conferencing system.

##### Perceived Involvement in Care [21]

Five participants completed the questionnaire about perceived involvement in care prior to the intervention. Almost all participants (80%) agreed that their healthcare provider gave them a complete explanation for their medical symptoms or treatment. Most participants also agreed (60%) that their healthcare provider encouraged them to talk about their personal concerns relating to their medical symptoms. In particular, most participants agreed (60%) that they asked their healthcare providers a lot of questions about their medical symptoms, including for recommendations (60%) about their symptoms. However, most participants disagreed (60%) that their provider asked them what they believe is causing their medical symptoms. The majority of participants also disagreed (80%) that they went into great detail about their medical symptoms.

Similarly, the majority (60%) of participants gave their opinion on the types of tests or treatment that their healthcare provider ordered, but most (60%) did not ask their healthcare provider to explain the treatment or procedure to them in greater detail. The majority of participants (60%) also disagreed that they suggested a certain kind of medical treatment to their healthcare provider. Two participants declined to respond to this item. Likewise, almost all participants (80%) disagreed that they insisted on a particular kind of test or treatment for their symptoms, with one participant declining to respond. All participants did not express doubts about the tests or treatment that their healthcare provider recommended.

Most participants (60%) disagreed that their healthcare provider asked them whether they agreed with the provider’s decisions. In particular, most participants disagreed (60%) that their provider encouraged them to give their opinion about their medical treatment.

##### Self-Efficacy [20]

Eleven participants completed the questionnaire about self-efficacy managing chronic disease prior to completing the online program. The mean score for all 11 participants who completed the questionnaire was 6.29 (SD = 1.78) with values ranging from 2 to 10. Participants were somewhat confident, on average, that they could keep the fatigue (mean = 6.09, SD = 2.12), dealing with emotional distress (mean = 6.45, SD = 1.86) and preventing other symptoms or health problems (mean = 6.45, SD = 1.57) from interfering with activities they wanted to do. In contrast, participants were less confident, on average, keeping physical discomfort or pain (mean = 5.82, SD = 1.78) caused by their disease from interfering with activities they wanted to do. Furthermore, participants were somewhat confident at completing tasks needed to manage their condition without professional assistance (mean = 6.64, SD = 1.36). In particular, participants were somewhat confident at dealing with tasks other than taking medication to reduce the impact of their condition on their everyday life (mean = 6.27, SD = 2.15).

#### Post-intervention Validated Surveys

##### Perceived Involvement in Care [21]

Ten participants completed the questionnaire about perceived involvement in care after the intervention. Almost all participants agreed that their healthcare provider gave them a complete explanation of their medical symptoms or treatment (90%). All participants agreed that their healthcare provider encouraged them to talk about personal concerns related to their medical symptoms, and the majority agreed that their provider encouraged them to give their opinion about medical treatment (70%). In particular, most participants agreed that their healthcare provider asked if they agreed with their decisions (60%) and what they believe is causing their medical symptoms (60%).

The majority of participants, with one participant declining to comment, also asked their providers to explain their treatment or procedure in greater detail (80%). Most participants asked providers a lot of questions about their medical symptoms (90%). Specifically, most asked for recommendations about their symptoms (90%). In contrast, no participant insisted on a particular kind of test or treatment for their symptoms or expressed doubts about the tests their healthcare provider recommended. However, almost all participants provided their opinion about the type of test or treatment ordered by their provider (90%). Only one participant suggested a kind of medical treatment to their provider (10%).

##### Self-Efficacy [20]

Ten participants completed the questionnaire about self-efficacy managing chronic disease after to completing the online program. The mean score for all 10 participants was 7.27 (SD = 1.66) with values ranging from 3 to 10. Participants were moderately confident, on average, that they could keep the fatigue (mean = 7.20, SD = 1.62) and physical discomfort or pain (mean = 7.60, SD = 1.71) caused by their disease from interfering with activities they wanted to do. In contrast, participants were less confident, on average, dealing with emotional distress (mean = 6.80, SD = 2.1) and preventing other symptoms or health problems (mean = 6.70, SD = 2.00) from interfering with activities they wanted to do. Furthermore, participants were moderately confident at completing tasks needed to manage their condition without professional assistance (mean = 8.00, SD = 1.15). In particular, participants were moderately confident at dealing with tasks other than taking medication to reduce the impact of their condition on their everyday life (mean = 7.30, SD = 1.16).

### Qualitative Data

#### Semi-structured Interviews

Findings from the semi-structured interviews are summarised in the following section. Refer to Table 1 for an overview of exemplar quotations by category.

**Table 1:** Exemplar quotes from the participants presented by category

Category	Quote	Participant
Patient lived experience	I was fortunate enough not to have any side effects.	P1
	And it turns out that I had some of the symptoms, but not nearly as many as I expected and not the ones that I was explained.	P3
	I found that I’m fairly clear of what I had to do	P4
	But generally speaking, I kind of look after myself, when I’ve got problems, common sense what should I do now, and then I do that.	P5
	I think when people are confronted. It is quite a shock and hence the million questions you have from oncologists and the literature.	P1
	Look, it was all happening very fast, from when I got diagnosed to when treatment started.	P4
	No. I’m living in a state of denial. Very few people know my diagnosis and no.	P2
	I have a couple of close friends who do know my diagnosis, who are both cancer patients, so that’s possibly helped in a way too.	P2
	They’re always forthcoming, people. And other than that, people like to help you.	P5
	The first time I came in…, the guy three things over, has a bad allergic reaction and is wheeled out in a wheelchair…	P4
	I see people there at the oncology place and they’re in a pretty bad way. You can see that they’ve got problems with it, but just luckily for me, I haven’t had huge problems	P5
Current approaches to health education	Get the information myself and then take what I don’t understand to my team and to the oncologist.	P1
	So had I been more challenged, perhaps I might have asked more questions or gone elsewhere.	P2
	…it was so much information that, I wasn’t aware of the likely effects on me personally.	P3
	We’re quite often given a lot of information very fast	P4
	There’s a lot of weird stuff out there as well, but there’s plenty of good stuff.	P5
	…They came and explained it all fairly well, one-on-one. I found all the instructions were fairly clear.	P3
	But then most of the typical thing, was they were basically oral conversations, where they’d come along and talk to me	P4
	I was given sheets of paper saying, listing possible side-effects of all the drugs they were going to put me on, and possible risks, and all like that, and outlining that.	P4
	And the handout material, or the brochures that they gave me were also pretty helpful.	P5
	…I did a certain amount of research online, bearing in mind I know what you read online is not necessarily true.	P2
	Nowadays the usual Google trip to see what my particular diagnosis would likely to lead to.	P3
Feedback on platform	I found it repetitive…And I don’t know if that was deliberate as part of the process for your research purposes. I don’t know. But I thought, “Oh here we go again”.	P1
	I didn’t feel threatened or demeaned by it or you know my self-esteem didn’t go down the toilet from it.	P1
	You know don’t answer them first thing in the morning when you wake up and let’s have a look at it	P1
	It wasn’t taking up all the time	P4
	I didn’t feel that I needed anyone to hold our hand through it, so I was quite independent in doing it.	P1
	I didn’t have to remember a password, or anything. I found that very easy.	P4
	I liked that the ease when you had to do the emailed questions, they were quick and easy.	P4
	I didn’t have any problems with accessing it at all. It’s good.	P5
	It was just tapping the email, and it went right to the site, sort of thing.	P4
	You could hit on it, and it could be done in three or four minutes, by the time we logged in, and read the questions, select the answer, and then hit submit. It was done, and it was quick and easy.	P4
Course content	My understanding is most of the programmes are how to manage your cancer, not how we can cure your cancer.	P5
	The information is good, generally common sense, isn’t it? What to do in certain situations.	P5
	The amount of information was pretty comprehensive.	P5
	Well initially, you see, not having one of those four things, I thought, “Where’s me? Where’s my weariness, et cetera?” Or, “Where are other things?”	P2
	No, I haven’t experienced anything that hasn’t been identified as one of the problems, a lot of things.	P3
	Admittedly, it seemed to be…I don’t know whether they’re meant to be, it seemed to be a lot of elderly people used in the photos.	P4
	I just had an issue with that. It’s just the way it was. It’s sort of led you to think differently to the most obvious answer.	P1
	People learn in so many different ways, you know? Yeah. People who see it well, so we’ll comprehend through texts people are more visual and auditory and things like that. And I think it’s good to have both	P1
	I get the feeling, and you didn’t say this in your answers, but this is a sort of influence that, look, if you’ve only got these don’t waste the doctor’s time. Sit tight until it either passes or it gets worse and then contact their doctors…	P5
Suitability of microlearning	I found it relevant, not probably enjoyable. I’d do it. It’s not anything you enjoy. You just do it	P4
	That was a good thing. It didn’t become a chore, the online side.	P$
	So, even though I got the answer right, I still looked up the answer.	P5
	I know the information’s out there but your way of presenting it, I think is good.	P5
	No, I would think it would be a benefit to most people.	P3
	I suppose they’re also probably very good, especially for some elderly people who may not be quite as…maybe have a bit of cognitive decline, or whatever.	P4
	I don’t know if you get people to agree to do that sort of thing or not. If they can’t be bothered or they forget.	P5
	They would have been to the clinics. They would have seen some of the people. So no, none of those cases would frighten them.	P2
Impact	In fact to me, I got the impression that it was leading you away from going to emergency more than anything else.	P1
	…, it made me more confident in what to expect and how to react to it.	P3
	It sort of clarified a lot of the questions, it did. And they’re the ones that I hadn’t had a specific answer to [continuing medication together].	P4
	It gave you a bit of guidelines how far it is. What might be normal, or what might be getting worrisome.	P4
	But if you’re a person who really can’t decide or a bit fearful about deciding, that information is very helpful.	P5
	…added to my knowledge of the sort of reactions that I could expect or should expect or the sort of things I should do.	P3
	It just reinforced how I should act under certain situations	P4
	I found it helpful, just reminding me what to look out for. If I was getting any symptoms, or anything, how I should act if I needed to.	P4
	…But it was good for me to think about what I would do in that situation, and to work out little strategies…	P2
Suggestions for improvements	I would have possibly included different options in the scenario. Apart from the correct one.	P1
	…things like meditation and what have you	P2
	…I don’t know how holistically people are thinking but I did make a comment in there that I would like to have training for the emotional side of things as well.	P1
	…They want to know if they’re going to lose their hair.	P2
	…Knowing that someone else is thinking what you’re thinking or feeling, how you’re feeling. It can be of great help and then there’s a support link to that	P1
	…In theory good, not so great in practice…A little bit of tweaking and I think you’d be getting to something	P1
	…I did find the questions sometimes a little repetitive and a little convoluted, but I don’t know, maybe it’s a round table discussion you need to have with other professionals.	P1
	I wondering actually, if you might have a second course further along? How long are people normally in treatment for this lung cancer business?	P2

##### Current Approaches to Health Education and Information Seeking

The majority of participants reported getting information about their condition and its management from their care team, usually by approaching them to ask a question. This was typically from their oncologist, but some participants also asked questions of nurses supporting them through chemotherapy. Generally, participants reported receiving information from the care team orally in conversation, though a number also reported being given pamphlets and other written handouts.

A small number of participants sought out information themselves, primarily online. One of these participants indicated they found information about their condition online, and then subsequently went to their care team for clarification if they did not understand something. Whilst participants reported some value in doing research about their condition online, it was also noted that this information was known to be potentially unreliable or untrue.

All the participants indicated that they received a lot of information about their condition quite quickly. One participant indicated they got a lot of information about their condition, and then they started therapy very quickly so there was a lot to take in. Information also came from a number of different sources, including a large amount of information from online sources.

##### Patient Lived Experiences with Cancer

All participants felt there were both an emotional and a physical toll of their diagnosis. A number of participants indicated that the initial diagnosis experience is quite confronting, and because a lot of information is provided to patients up front it could be quite scary as it implied the side effects were going to happen. It was also noted that not being able to cope emotionally can have a wide-reaching effects, and it is easy to feel isolated during therapy as if you are the only person going through the experience.

Most participants indicated they had some level of support system from family and friends whilst undergoing therapy. One participant indicated that whilst people had good intentions and wanted to support you, they could give unusual opinions and advice. One participant indicated that they had not told many people about their diagnosis.

The participants indicated that there is considerable variation in the experience of individual patients during therapy. Some patients have major side effects of treatment, and others may witness patients having a difficult time with their therapy which can make their own experiences more emotionally burdensome. One participant mentioned that there is also variation in how individual patients approach therapy, with some viewing it as a curative exercise and others more focused on management of disease.

##### Existing Self-Efficacy

The majority of interview participants indicated that they felt they had reasonable self-efficacy managing their disease prior to undertaking the program. One participant indicated they felt they were a generally independent person who was used to looking after themselves, and this translated into confidence managing side effects of cancer therapy.

##### Treatment Side Effects

The majority of interviewees indicated that they had experienced very few or no side effects as a result of their chemotherapy. One participant reported experiencing nausea, and another suggested they felt a bit off each day. Several participants who had not experienced side effects of chemotherapy felt that it was inevitable they would eventually experience them, and there was an expectation that side effects were party of receiving therapy.

##### Applicability of Course Content

The majority of interviewees thought the side effect categories covered in the program were relevant. A number of participants indicated the content was good, generally relevant and not overly complicated. Some participants indicated the content seemed to describe fairly obvious side effects, with common sense answers. Two participants also indicated they did not quite feel they could ‘see themselves’ in the content, either because the side effects described did not align with their experiences or because the images used in cases did not look like them.

Interviewees indicated that they felt some of the case content could be convoluted or challenging to follow. A number of interviewees also disagreed with the answers that were considered ‘correct’ for individual cases. This was particularly common for cases where the answer was to self-manage a side effect, when the participant felt the side effects described were severe enough to warrant going to an emergency department.

##### Suitability of Microlearning for Patient Health Education

All interviewees felt the microlearning program was engaging and a good thing to complete when undergoing therapy. One participant reported looking forward to receiving cases for the question. A number of interviewees indicated they liked the way the microlearning presented information on side effect management, and it was useful for accessing information event though participants knew the information was available elsewhere. Two participants indicated that they reviewed the reference material in the cases and did additional research as a result of receiving a case from the microlearning platform. One participant felt that the platform had limitations as it could cases had to either be right or wrong.

The majority of participants felt that the microlearning program would be beneficial to other patients. One participant was unsure if the program would be easily accessible to an older population of patients.

When asked about whether the content and delivery of it via the microlearning platform was confronting, all interviewees said no. Participants did not think there would be any way that the program could be distressing or confronting. One interviewee noted that the biggest point of distress with a cancer diagnosis was at the initial content. Two others suggested that after undergoing a few sessions of chemotherapy the trepidation of therapy decreases. No participants had any concerns about potential harm or distress that would occur as a result of completing the microlearning program.

##### Impact of the Program on Knowledge and Confidence

The majority of interviewees indicated that the online program had a positive impact for reinforcing their existing knowledge about side effects of chemotherapy. Although most participants did not experience side effects during the intervention period, a number felt that if they had the content in the course would have provided them useful information on side effect management.

Two participants indicated the program was particularly impactful for helping generate new knowledge on coping strategies for chemotherapy side effect management. This was considered important as participants expected they would experience side effects at future points in their therapy, and that the strategies learnt in the program would be applicable for managing them.

Regarding improving confidence self-managing side effects of chemotherapy, interviewees had mixed opinions. A number of participants indicated that the course made them feel confident what to expect as their chemotherapy progressed, and how to manage side effects when they did occur. Two interviewees felt that the course was leading them away from going to emergency in preference for self-management. However, another two participants felt that the course helped build confidence about distinguishing between mild side effects that could be self-managed and those that warranted contacting the treatment team or getting professional support.

Participants demonstrated considerable retention of content during the interviews. Although the interviews were not designed to explore knowledge retention, all participants cited examples of cases from the program when providing feedback on the intervention. One participant could cite the number of cases in the program, and another could recall the answers to individual cases.

##### Suggestions for Improvement

Interviewees were asked to provide suggestions on how to improve the program if it was to run in future. The majority of suggestions related to including specific content in the program, particularly around emotional supports for patients undergoing therapy. One interviewee made a general comment that the program was generally good, but the content required further tweaking to improve question clarity. Another interviewee suggested the program could be enhanced by sharing patient journeys or somehow indicating that patients were not alone on their journey.

Two interviewees indicated there was value in an additional program that described what to expect when they had progressed further through therapy. There was interest in a program exploring how long treatment continued for the ‘typical’ patient, and also when side effects would occur if they were not experienced early on.

#### Symptom Diary

Three symptom diaries were returned that indicated participants generally experienced few symptoms of low severity. Only one participant reported experiencing pain of average to high severity periodically over 10 weeks. Two diaries were partially completed with 3 and 28 days missing, respectively.

All participants only sought professional help when the severity of each symptom deviated from their baseline severity. For example, one participant sought help for breathlessness when they started experiencing the symptom whilst another participant only sought professional help when the severity of pain increased from very low or low severity to average and high severity. Almost all medical visits or interactions were accompanied by a pharmaceutical intervention such as increasing or changing the dose of an existing medication or prescribing a medication to treat the symptom.

#### Free-Text Post-intervention Survey

A total of 10 participants completed the online survey post-intervention. Most participants agreed (50%) or strongly agreed (20%) that taking the Qstream program online was enjoyable. Most participants also agreed (60%) or strongly agreed (30%) the program was easy to use. In particular, all participants agreed (70%) or strongly agreed (30%) the multiple-choice format was easy to use. Participants also mostly agreed (60%) or strongly agreed (30%) that the Qstream cases were easy to understand.

Majority of participants agreed (60%) or strongly agreed (20%) that the examples of symptoms and symptom management in the program were relevant to their treatment plan. Furthermore, most participants agreed (60%) or strongly agreed (30%) that the cases increased their confidence around symptom identification and management. Participants also mostly agreed (30%) or strongly agreed (60%) the program increased their confidence to identify symptoms that may occur because of their cancer treatment. Similarly, most participants agreed (30%) or strongly agreed (50%) the program would increase their confidence self-managing possible symptoms of their cancer treatment. This included agreeing (40%) or strongly agreeing (50%) that the program increased their confidence knowing when to seek expert help for symptom management.

All participants either agreed (20%) or strongly agreed (80%) that they completed the program on their own. Conversely, almost all participants either disagreed (20%) or strongly disagreed (70%) that they needed help from family, friends, carer or cancer nurse to complete the program. Participants also mostly disagreed (10%) or strongly disagreed (70%) that they completed the course with the support of a family member or friend. Similarly, most participants disagreed (10%) or strongly disagreed (80%) that the completed the majority of the course with a member of the healthcare team for support.

Most participants agreed (20%) or strongly agreed (40%) that they would complete another Qstream program if offered. One participant strongly disagreed that they would complete another program (30%), whilst three participants expressed no opinion (30%).

Analysis of free-text responses to the post-intervention survey showed that several participants found the program informative (30%) and relevant (30%) to their cancer experience. One participant noted the program would be adaptable to most patients. Another participant found the program improved their understanding.

One participant cautioned that new patients may be overwhelmed by the amount of information, whilst another participant noted patients may respond differently to the question depending on the severity of the symptom.

Finally, analysis of free-text responses from the ten respondents to the post-intervention survey indicated that all participants provided further feedback about the Qstream program. Half of the participants found the program easy to use, with one participant commending the email format. Participants had mixed responses about the suitability of microlearning, with two participants finding the repetition unnecessary, whilst one participant appreciated the opportunity to reattempt questions. Three participants liked the Qstream program with no suggestions for improvements. Other participants recommended including more questions on other symptoms and different types of tests or treatment, greater detail in the feedback and a refresher course as patients progress through treatment.

## Discussion

This study findings indicated that the microlearning platform could be used for delivering this type of education to patients. Most patients found the platform an engaging way to access information and useful to complete whilst undergoing chemotherapy. The program had a completion rate about 70%, which indicates a relatively high retention rate for an online learning intervention [23]. Although the platform worked for delivering the health education, study findings suggested that the multiple-choice format of questions could be problematic as the format required patients to choose a right or wrong response which may not align with their lived experience. A secondary aim of the study was to understand the perspectives of patients with lung cancer regarding the acceptability of microlearning as a mechanism for delivering health education. Findings from the study indicated that the concept was broadly acceptable, with most participants indicating that the program was enjoyable to complete and some also indicating that the content was also informative and relevant. Another secondary aim of the study was to understand the potential impact of microlearning on patient self-efficacy, knowledge and confidence managing side effects of chemotherapy. Study findings indicated that the majority of participants felt the online program had a positive impact for reinforcing their existing knowledge about side effects of chemotherapy. Regarding patient self-efficacy managing their chronic disease, participants reported an improvement managing side effects of chronic disease and confidence dealing with tasks to reduce the impact of their condition on everyday life post-intervention, though it should be noted only a small number of participants responded to the self-efficacy questionnaire pre- and post-intervention.

Findings from the study described in this manuscript suggested that lung cancer patients often receive a considerable amount of information about their condition. The information provided to patients is also disseminated quite close to when a patient receives their initial diagnosis and start therapy, which can lead to feelings of information overload. This finding aligns with the existing literature which shows that patients can feel overwhelmed by the amount of information they received about their chronic condition, particularly patients with a cancer diagnosis [24]. Findings from our study suggest one of the advantages of delivering health information to patients utilising the microlearning platform is that it spaces content delivery over a number of weeks. This spaced approach to delivering health information to cancer patients has the potential to reduce the perception of information overload soon after diagnosis or close to when chemotherapy is being started. Further, the spaced approach of microlearning may prove effective for prompting patients to seek health information from their treatment team progressively across their therapy period, when they encounter content in the online course, rather than at the start.

Study findings also suggested patients pro-actively sought out guidance about chemotherapy side effects, when they felt the information was required. The primary source of information was from the healthcare team in the cancer centre. Findings showed that patients felt the healthcare team was available to answer questions about their chemotherapy whenever questions arose. This may explain why most participants in the study perceived a high level of care involvement and self-efficacy, as it has been shown that high involvement in care is a factor that enhances perceived self-efficacy in patients [10, 11]. Interestingly, study findings suggest a portion of patients are seeking out information online about management of their condition and side effects of chemotherapy. This broadly aligns with the existing literature which suggested that cancer patients, like many other patient groups, engage in information seeking online [16]. Although some participants engaged in online information seeking, findings also indicated they were quite critical of the information and perceived online information as being variable in quality.

Findings related to the content of the microlearning program suggested participants had varied perspectives on its relevance to their individual therapy experiences. Whilst some participants found the side effect categories the content focused on appropriate and the relevant, other participants felt the content was too obvious and did not align closely enough with their lived experience. The content was designed to cover the most commonly experienced clusters of chemotherapy side effects [5], but participants in the study frequently reported not experiencing side effects during their therapy. It is possible the low incidence of chemotherapy side effects reported by participants could explain the variation in feedback on course content, as many participants did not need the information in the microlearning cases. Some support for this explanation is found in the returned symptom diaries which indicated participants experienced no to low-severity symptoms and sought professional help immediately when severity increased. Study findings also suggested considerable interest in developing microlearning content related to the emotional supports for patients undergoing chemotherapy. Although it did not emerge in the participant feedback, there would likely be considerable value in developing content that is culturally relevant for delivery via microlearning. The study was undertaken in a population with a large number of CALD patients, and a need to develop health information that is culturally relevant for this population has been noted in the literature [15].

A final interesting study finding related to personalisation of the content. Study participants reported a desire to ‘see themselves’ more clearly in the content of the cases. The literature has shown that personalisation of microlearning to align with clinical practice is of interest to health professionals [25] but there is a dearth of research on personalisation of online health education for patients. Findings from the research described in this manuscript suggest this type of personalisation may be relevant for patient health education as well. Further, study findings suggest there is not just a need to personalise content and dissemination strategies to individual patients, but there may be opportunities to use authentic scenarios to explore the patient journey and share stories of different cancer patients.

### Limitation

A limitation of the study is that it was not possible to recruit the target number of participants in order to demonstrate a significant change in response to the validated questionnaires administered pre- and post-intervention. Study recruitment had to conclude in response to the COVID-19 pandemic, which is why the sample size is smaller than originally intended. Participants were not individual tracked in pre- and post-responses to the validated questionnaires, which meant data could only be presented descriptively by cohort not individually. The intervention also used digital technology to disseminate health information in English, for a population where there is a proportion of patients without high levels of access to technology or reliable internet and a high level of CALD patients. This may have resulted in the intervention not being undertaken by patients who would have obtained benefit from it.

Future researchers should further explore the value of digital platforms that can space the delivery of health information for cancer patients in order to reduce information overload close to the point of diagnosis. Furthermore, there is considerable scope to investigate the content of such courses so that it is most relevant to the needs of individual patients, and whether delivery of content can be personalised to be delivered at a time and in a format that suits the individual patient’s needs.

## Conclusions

Patients with a diagnosis of cancer receive a large amount of information about managing side effects of chemotherapy. This information can be overwhelming, particularly when provided in a single bundle near the time of diagnosis or initiation of therapy. Microlearning is an online learning method that spaces distribution of content over several weeks and repeats content to reinforce learning. It has potential as a method of delivering health education to cancer patients undergoing chemotherapy. However, for the method to be most effective, content of cases needs to be designed to closely align with the experiences of patients completing the program. There is also a need to design microlearning content to allow patients to provide a range of responses to content, rather than binary right or wrong answers.

## Data Availability

The datasets used and/or analysed during the current study are available from the corresponding author on reasonable request.
